# Innovative strategies for minimizing hematoma risk in MRI-guided breast biopsies

**DOI:** 10.2478/raon-2025-0004

**Published:** 2025-01-22

**Authors:** Michael P Brönnimann, Matthew T McMurray, Johannes T Heverhagen, Andreas Christe, Corinne Wyss, Alan A Peters, Adrian T Huber, Florian Dammann, Verena C Obmann

**Affiliations:** 1 Department of Diagnostic, Interventional and Pediatric Radiology, Inselspital, Bern University Hospital, University of Bern, Bern, Switzerlandmichael.broennimann@charite.de; 2 Department of Radiology, Charité – Universitätsmedizin, Berlin, Berlin, Germany; 3 Department of Nuclear Medicine and Radiology, Cantonal Hospital Lucerne, Lucerne, Switzerland

**Keywords:** biopsy, interventional, image-guided biopsy, magnetic resonance imaging, breast neoplasms, female, hematoma

## Abstract

**Background:**

The study aimed to investigate the reduction of hematoma risk during MRI-guided breast biopsies by evaluating position-dependent intervention parameters and characteristics of the target lesion.

**Materials and methods:**

We retrospectively analyzed 252 percutaneous MRI-guided breast biopsies performed at a single center between January 2013 and December 2023. Two groups were built depending on the severity of relative hematoma formation (using a cut-off ≤ 7.62 cm^3^ or > 7.62 cm^3^). Potential influencing variables were assessed, such as patient demographics, interventional parameters related to anatomical landmarks, and lesion characteristics. Fisher’s exact test and Mann-Whitney-U-Test were used to calculate the statistical difference between groups of categorical, dichotomous, and continuous variables. Multivariable logistic regression was used to identify the strongest association with relative hematoma formation.

**Results:**

The univariate analysis showed that relatively larger hematoma occurred significantly more frequently when the patients were younger (P = 0.002), the relative distances from the target lesion to the nipple (P = 0.001) as well as alongside the access path (P = 0.001) were greater and when the vacuum-assisted biopsy system was used in contrast to the Spirotome® (P = 0.035). Multivariable logistic regression analysis also showed that these were independently associated with the occurrence of relatively larger hematomas. Epinephrine in the local anesthetic, lesion location classified by specific quadrant, and pathological findings did not influence the extent of the hematoma.

**Conclusions:**

Our findings underscore the importance of strategic procedural planning to minimize hematoma occurrence and enhance patient safety during MRI-guided breast biopsy procedures.

## Introduction

MRI is more sensitive than alternative imaging methods like ultrasound or mammography for women at elevated risk of breast cancer, detecting over half of lesions exclusively on MRI.^1,2^ It can detect primary cancer in patients suspected of having occult breast cancer.^3-5^ As per the guidelines of the American Cancer Society (ACR) and the European Society of Breast Imaging (EUSOBI), MR-guided biopsy is recommended for suspicious lesions exclusively detected by MRI (referred to as MR-only lesions).^6-8^

To minimize sampling errors and not underestimate the target lesions, percutaneous, stereotactic large core biopsies larger than 11 gauge, either with vacuum-assisted devices or with manual devices such as the Spirotome®, have prevailed over core biopsies.^9-13^ Hematoma is identified as the primary complication of this procedure across different contexts, with the incidence of mammographically evident hematoma reported to be as high as 45%.^14-19^ While many hematomas do not require additional intervention, the clinical significance of existing studies remains unclear.^15-20^ Furthermore, the study situation is also very inconsistent in recording hematomas. Most reports attempted to record these by external observation *e.g*.^21^ or indirectly *e.g*.^19^ To date, no intraprocedural factors have been identified to reduce the risk of hematoma occurrence in MRI-guided breast biopsies.

A decisive aspect in developing hematomas could be the asymmetrical blood supply to the breast. Thus, 60% of the breast is supplied by perforating branches of the internal thoracic artery, which lies on the medial side.^22^ Of this, at least 60% is specifically provided by the superomedial perforators.^23^ Furthermore, the internal thoracic artery mainly supplies the nipple-areola complex (NAC).^24^ The prone position during MRI-guided breast biopsy could aggravate this. Also, the growth and advancement of breast cancers are associated with heightened neovascularization.^25^ We, therefore, hypothesize that larger hematomas are more likely to occur in the upper and outer quadrants near the NAC in malignant lesions.

The study aimed to investigate the hematoma risk during MRI-guided breast biopsies by evaluating position-dependent intervention parameters and characteristics of the target lesion.

## Materials and methods

The study received approval from the Ethics Committee of the Canton of Bern (BASEC Project-ID 2024-00805) and adhered to the principles outlined in the Declaration of Helsinki. The authors had complete access to the data and assumed full responsibility for its integrity. Written informed consent was obtained from all patients.

### Study population

This study retrospectively analyzed 306 percutaneous MRI-guided breast biopsies conducted at our university hospital between January 2013 and December 2023 in 300 women with thus 6 women who underwent more than one biopsy.

**FIGURE 1.**
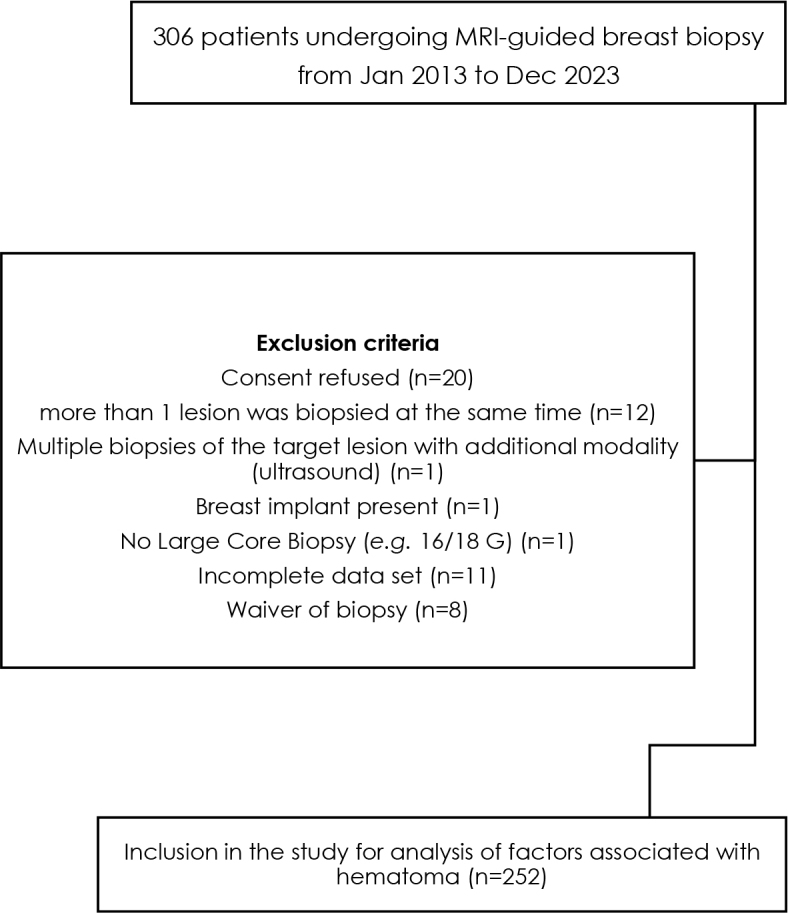
Flowchart shows the study population.

The exclusion criteria were defined consecutively to avoid possible bias due to non-physiological local conditions (breast implant; more than one lesion was biopsied at the same time; multiple biopsies of the target lesion with additional modality) or rare intervention techniques (no large core biopsies *e.g*. 16 or 18 G and to homogenize the groups (Figure 1)).

Two groups were formed based on the severity of the relative hematoma volume. The severity was categorized dichotomously according to whether the measured relative hematoma volumes were above or below the mean value.

### Baseline evaluation and biopsy technique

Before the biopsy, all patients underwent a clinical evaluation, including a thorough medical history review and standard blood tests. The procedure required an INR value below 1.5 or a Quick value above 60%, an Hb value exceeding 80 g/L, and a platelet count of over 50 × 10^9^/L. These blood values must not be older than 5 days. According to our guidelines, NSAIDs (non-steroidal antiinflammatory drugs) and clopidogrel had to be stopped 5 days, heparin 6 hours, rivaroxaban 1 day, dabigatran and endoxaban each 3 days before intervention. Eight interventionalists, each with more than 5 years of experience, performed the biopsies. Contraindications for MRI, such as pregnancy, inability to lie prone for 60 minutes, or the administration of contrast medium containing gadolinium, were explicitly requested in advance in the questionnaire.

**FIGURE 2.**
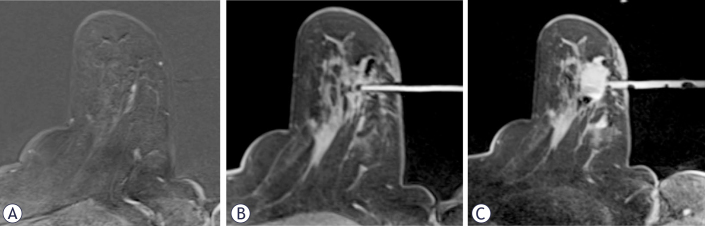
MRI-guided breast biopsy with hematoma as a sequela. **(A)** Target lesion in the lower outer quadrant. **(B)** Biopsy needle in target position. **(C)** After vacuum-assisted biopsy, a hematoma has formed. Within it, a recognizable susceptibility artifact is due to the marker.

All biopsies were conducted utilizing a 1.5 T Siemens Magnetom Aera/SolaFit (Siemens Healthinieers, upgrade of the machine performed in 06/2022) paired with a dedicated four-channel open breast coil (Invivo Interventional Instruments, Wurzburg, Germany) and a needle positioning add-on device (Noras, Germany). Patients were positioned in the prone position on the MRI table with the affected breast compressed within the biopsy device to minimize motion during the procedure. Imaging was performed before and after administering 0.1 mmol/kg of contrast agent (Dotarem, Guerbet, France) using a T1-weighted dynamic contrast-enhanced subtraction sequence lasting 103 seconds. This imaging protocol provided coverage of the breast with nearly isotropic voxels (slice thickness 1 mm, Repetition Time (TR) of 7.62 (milliseconds) ms, Time to Echo (TE) of 4.77 ms), facilitating image reconstruction in any plane. Either the grid or pillar and post system was used. The specialized breast biopsy planning software (Siemens) was utilized to acquire lesion coordinates and guide needle positioning. The needle path was planned according to our best practices to ensure the shortest distance from the skin to the lesion while avoiding larger vessels and maintaining a safety margin from the skin. After the initial imaging, the table was retracted from the bore to facilitate disinfection and the administration of local anesthesia. Lidocaine 1% (maximum 20 ml, Streuli Pharma AG, Uznach, Switzerland), was administered to the skin, subcutaneous tissues and deep. The interventionalist could choose whether to give the one mixed with epinephrine (Lidocain-Epinephrin 2% Streuli, Streuli Pharma AG, Uznach, Switzerland). A small incision was made in the skin to facilitate the smooth entry of the device. The introducer stylet, was inserted through the needle guide to the predetermined depth. Subsequently, the stylet was withdrawn and replaced with a sterile plastic MRIvisible obturator. T1-weighted fat-saturation images were then acquired to confirm the depth and position of the introducer. Either a large volume biopsy with a manual device Spirotome® 8 gauge (G) (Bioncise, Wellen, Belgium) and max. 3 samples or a Vacuum-assisted biopsy (VAB) with an EnCore® (BD, New Jersey, USA) 7G/10G and max. 24 samples was done. After the marker (SenoMarkTM, BD, New Jersey, USA) was placed, a control sequence T1 and T2, again technical details were performed (Figure 2). The breast was compressed manually for 15 minutes followed by a compression bandage for the next 24 hours.

### Data collection

All procedures were reviewed by a board-certified interventional radiologist with eight years of experience and a radiology resident with four years of experience. Neither of them performed any of the interventions, and all were blinded to the patient`s medical history.

The perpendicular distance from the pectoralis major muscle to the lesion (in mm) was recorded with the consideration of indirectly measuring the effect of gravity. Other patient- and techniquerelated variables assessed on the interventional MRI images included interventional date, birth date, age of the patient (in years), procedure time (in minutes), target lesion size (in mm), biopsy side, biopsy angle, distance lesion to nipple (LN in mm), access path length measured along the needle from the skin to the lesion (in mm), lesion location according to the breast quadrants, breast and hematoma volume (cm^3^). All volumes were manually obtained from the multiplanar reconstruction (MPR) of the three-dimensional (3D) T1-weighted sequence with spectral attenuated inversion recovery (SPAIR) acquisition. The manually measured hematoma volume and absolute distances were set in relation to the breast volume, as we ultimately rated this as the most meaningful in the overall context. For this purpose, we identified the mean of the relative hematoma volume of all measured volumes (7.62 cm^3^) and defined this as the cut-off.

**FIGURE 3.**
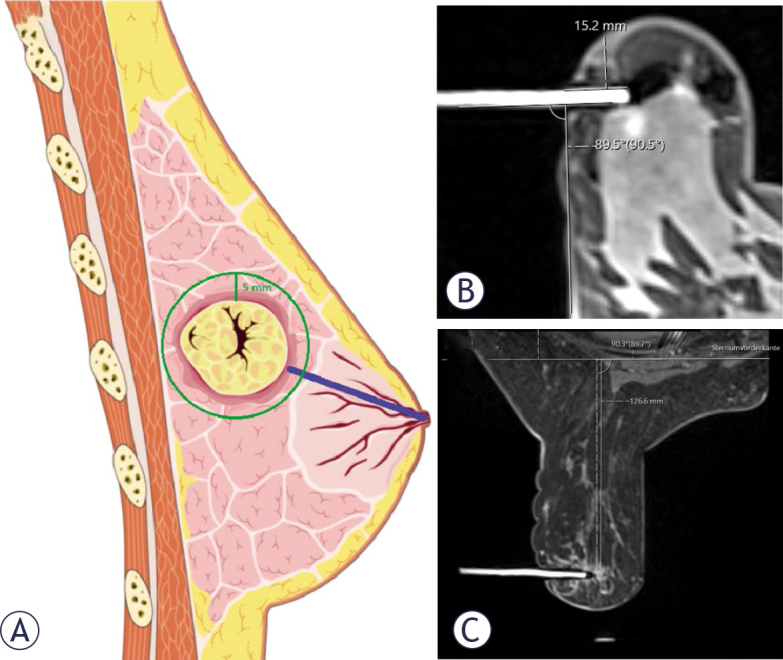
Illustration of the measurements. **(A)** In blue, the distance from the lesion to the nipple. A circle was drawn around the target lesion to capture the proportion of the surrounding mammary gland. These measurements were taken after multiplanar reconstruction (MPR) of the dynamic T1 subtraction sequence. **(B)** Access route and biopsy angle. **(C)** Perpendicular distance from the pectoralis major muscle to the lesion in T1-weighted with spectral attenuated inversion recovery (SPAIR) sequence.

To evaluate the possible influence of surrounding mammary gland tissue on hematoma formation, a 5 mm larger circle was drawn around the target lesion to assess the amount of surrounding mammary gland tissue. The quarter rule was used for this purpose (Figure 3).

A Sectra workstation was used to review the images (IDS 7, version 24.2, 2022, Linköping, Sweden). We recorded the size of the biopsy system, type of local anesthetic and number of samples from the intervention report. The histological results from the target lesion and the patient`s history after intervention were also collected retrospectively from the electronic medical record. The following pathological reports were categorized into benign, high-risk lesions and malignancy. High-risk lesions were defined according to Heller *et al*.^26^ as atypical ductal hyperplasia, lobular intraepithelial neoplasia, lobular carcinoma in situ, papilloma, atypical lobular hyperplasia, radial scar, and flat epithelial cell atypia.

### Statistical analysis

We utilized IBM SPSS Statistics for Windows, version 28 (IBM, Armonk, NY), for all statistical analyses. Univariate analysis was conducted using Chi-Square and Fisher exact tests for categorical variables, and the Mann-Whitney-U-Test for continuous variables, with a significance level set at P < 0.05. The Kolmogorov-Smirnov test was used to test for normal distribution. Multivariable logistic regression was used to assess potential confounders and risk factors for the development of hematoma, with model goodness of fit evaluated using the Hosmer-Lemeshow test. To prevent overfitting, we adhered to the rule of ten by including five independent variables in the multiple logistic regression model, selected based on their significance or proximity to significance, while considering a minimum group size of n ≥ 25 for categorical predictors. Therefore, we included the procedure time for the regression. The analysis was overseen by a senior statistician from the Clinical Trials Unit (CTU) of the Faculty of Medicine at the University of Bern.

## Results

### Study population

**FIGURE 4.**
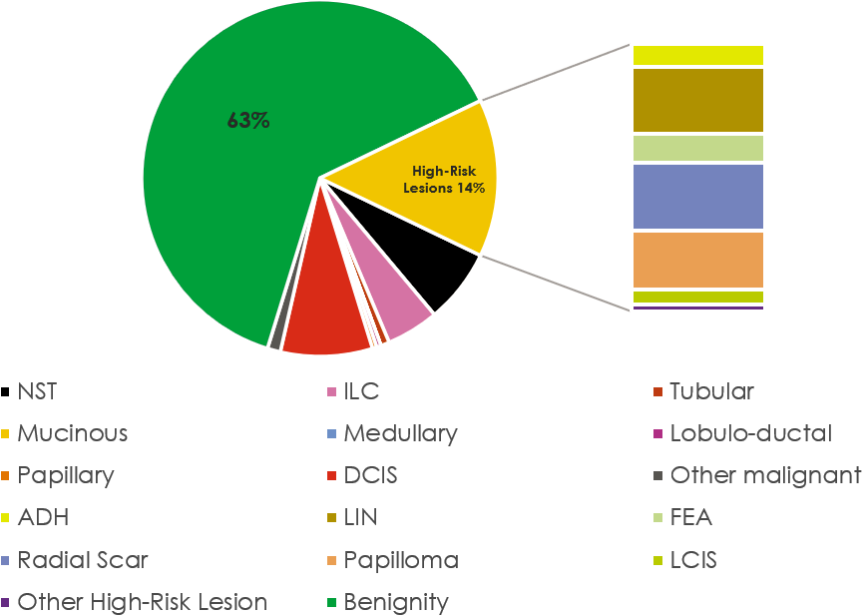
Histological findings of breast biopsies. ADH = atypical ductal hyperplasia; DCIS; ductal carcinoma in situ; FEA = flat epithelial cell atypia; ILC = invasive lobular carcinoma; LCIS = lobular carcinoma in situ; LIN = lobular intraepithelial neoplasia; NST = non-special type

A total of 252 biopsies met the inclusion criteria, with a mean patient age of 51.96 ± 11.69 years (range 22–84 years). All variables in both groups were not normally distributed, as indicated by the Kolmogorov-Smirnov test (P < 0.001). There were no significant differences in pathological findings or their locations between the two groups. The majority of biopsied lesions were benign (63%, Figure 4), located in the outer quadrants of the breast (62%), and surrounded by 0–25% mammary gland tissue (42%). Slightly more than a quarter (26%) of the biopsied breasts had been previously treated (Table 1).

**TABLE 1.** Univariate analysis for relatively larger hematoma with patient demographics and lesion characteristics

Survey of breast biopsies							
Parameter	All (n=252)		Relatively smaller hematoma (n = 200)		Relatively larger hematoma (n = 52)		*P* Value
**Age (y)**	51.96	±11.69	53.09	±11.09	47.63	±12.984	0.002*
**Procedure time (min)**	41.83	±13.39	42.58	±13.80	38.96	±11.324	0.072
**Right biopsy side**	117	46%	90	45%	27	52%	0.436
**Number of samples**	12.08	± 4	11.81	±4.08	13.1	±3.533	0.243
**Biopsy system**							0.035*
VABB	228	90%	177	88.5%	51	98%	
Spirotome	24	10%	23	11.5%	1	2%	
**LAE**	171	68%	133	67%	38	73%	0.408
**Lesion size (mm)**	11.2	±6.612	11.27	±6.55	10.94	±6.89	0.425
**Distance LN in RBV**	0.079	± 0.05	0.07	± 0.04	0.11	±0.04	0.001*
**Access path length in RBV**	0.055	± 0.046	0.05	± 0.03	0.08	±0.07	0.001*
**PDPectLesion in RBV**	0.065	± 0.036	0.06	± 0.04	0.07	± 0.04	0.143
**Biopsy angle (degree)**	90.79	± 10.4	90.93	± 9.55	90.27	± 13.26	0.443
**Pathological findings**							0.831
Benignity	159	63%	125	63%	34	65%	
highrisk lesions	36	14%	28	14%	8	15%	
Malignancy	57	23%	47	24%	10	19%	
**Pretreatment**	65	26%	55	28%	10	19%	0.286
**Lesion location**							0.399
Upper inner q	47	19%	34	17%	13	25%	
Upper outer q	84	33%	64	32%	20	38%	
Lower inner q	24	10%	21	11%	3	6%	
Lower outer q	72	29%	61	31%	11	21%	
Areolar	25	10%	20	10%	5	10%	
**Proportion of mammary gland**							0.215
0–25%	105	42%	85	43%	20	38%	
25–50%	48	19%	37	19%	11	21%	
50–75%	47	19%	37	19%	10	19%	
75–100%	52	21%	41	21%	11	21%	

1 Unless stated otherwise, data are number of biopsies. ± standard deviations. X2 (R X 2), Fisher’s exact test and Mann-Whitney-U-Test were used to calculate the statistical difference between groups of categorical, dichotomous, and continuous variables, respectively. Data are mean ± standard deviation.

1 * Statistically significant (defined P < 0.05)

1 LAE = local anesthesia mixed with epinephrine; LN= lesion to nipple; min = minutes; mm = millimeters; PDPectLesion = perpendicular distance from the pectoralis major muscle to the lesion; Pretreatment = affected breast pretreated; RBV = in Relation to Breast Volume; VABB = vacuum-assisted breast biopsy; q = quadrant; Y = Year

### Hematoma formation during MRI-guided breast biopsy

On average, the MRI-guided breast biopsy lasted 41.9 minutes. In 90% of cases, the biopsy was performed as a VAB, with the majority of cases preceded by the use of local anesthetic mixed with ephedrine (68%). A hematoma occurred in 70% (178/252) of cases, with a hematoma volume of ≥14 cm^3^ (diameter of 3 cm) detected in 6%, and a volume of ≥ 33.5 cm^3^ (diameter of 4 cm) detected in 0.8%. The univariate analysis showed that relatively larger hematoma occurred significantly more frequently when the patients were younger (P = 0.002), the relative distances from LN (P = 0.001) as well as the access path (P = 0.001) were larger and when the VAB instead of a Spirotome system was used (P = 0.035). Both relatively smaller hematomas (11.5% *vs*. 88.5%) and relatively larger hematomas (2% *vs*. 98%) occurred significantly less with the Spirotome than the VAB. We did not observe a significantly increased frequency of relatively larger hematoma depending on epinephrine in the local anesthetic (P = 0.408), lesion location classified by specific quadrant (P = 0.399), lesion size (P = 0.425), biopsy angle (P = 0.443), the perpendicular distance from the pectoralis muscle to the lesion (P = 0.143), adjacent glandular tissue (P = 0.215) or histopathological result (benignity, P = 0,749; highrisk lesions, P = 0.581, malignancy, P = 0.825).

### Association of lesion characteristics and technical parameters with the occurrence of relatively larger hematoma

Multivariable logistic regression analysis showed that lower age (OR 0.969, 95% CI 0.934 –1, P = 0.048), the use of a VAB system (OR 11.798, 95% CI 1.341– 103.8, P = 0.026), the LN distance (P < 0.01) and access path length (P = 0.029) were independently associated with relatively larger hematomas (Table 2). A good model fit with an R^2^ = 0.222, P < 0.001. Cohen’s f2 is 0.28, corresponding to a good medium effect.^27^

**TABLE 2.** Results of multivariate logistic regression analysis for relatively larger hematoma

	B	S.E.	Wald test	df	*P* value	Odds ratio	95% CI	
Variable							-	+
Age (y)	-0.031	0.160	3.927	1	0.048*	0.969	0.94	1
Biopsy system	2.468	1.11	4.947	1	0.026*	11.798	1.341	103.811
Distance LN in RBV	9.501	3.538	7.214	1	0.007*	13379	13.037	13.7 × 10^6
Access path length in RBV	7.622	3.49	4.77	1	0.029*	2043	2.186	1.9 × 10^6
Procedure time (min)	-0.025	0.015	2.759	1	0.097	0.975	0.947	1.005

1 The total number of cases in the cohort for the multivariate analysis was n = 252

1 B = Regression coefficient; CI = Confidence interval; df = Degree of freedom; LN = Lesion to nipple; min. = minutes; RBV = in relation to breast volume; S.E. = standard error;

1 * statistically significant (defined P < 0.05); Y = years

## Discussion

This study aimed to assess factors influencing hematoma risk in MRI-guided breast biopsies. Through retrospective analysis of 252 cases, patients were stratified by hematoma size, and various parameters including patient demographics, anatomical features, and lesion attributes were scrutinized. Univariate analysis unveiled significant associations between larger hematoma formation and younger patient age (P = 0.002), increased LN distance (P = 0.001) and access path length (P = 0.001), and utilization of VAB instead of Spirotome® (P = 0.035). Multivariable logistic regression confirmed these associations as independent predictors of relatively larger hematomas, while factors such as epinephrine in local anesthetic, lesion location classified by specific quadrant, and pathological findings did not influence hematoma extent. Overall, hematoma incidence was observed in 70% of cases. The results suggest that asymmetric blood supply to the breast supply and the gravitational force play a less significant role in the development of hematomas during MRI-guided breast biopsies than previously anticipated.

Our study findings align with prior research. Kettritz *et al*.^28^ documented 0.87% incidence of large hematomas (≥4 cm in diameter), while our study observed a rate of 0.8% (relative to a volume of 33 cm^3^). Perlet *et al*.^29^ reported 1.75% occurrence of hematomas with a size of ≥3 cm. In contrast, our study recorded a 6% incidence, slightly higher likely due to differing follow-up protocols. In instances of uncertainty, they conducted repeat MRI scans 24–48 hours post-biopsy. Our direct comparison between two biopsy modalities, VAB and Spirotome®, corroborates previous research. Hematoma occurrence rates were reported to be as high as 45% after VAB and 16% following large core biopsy with the Spirotome®.^14–19,30^ This discrepancy is likely attributed to the increased traumatic injury associated with VAB, regardless of the sample size. Consequently, our study identified a significantly higher incidence of relatively larger hematomas associated with VAB (P = 0.035), a finding further supported as an independent predictor in multivariable logistic regression analysis.

Our investigation indicates that gravitational forces do not exert a significant influence on hematoma development in MRI-guided biopsies, as evidenced by the absence of a significant occurrence of larger hematomas despite a greater relatively perpendicular distance from the pectoralis muscle to the lesion (P = 0.143). While it is established that lesion dimensions are overestimated and exhibit a higher wash-in peak in the prone position compared to the supine position^31^, McGrath *et al*.^32^ observed that breast compression during MRIguided breast biopsy may diminish perfusion and result in inadequate parenchymal enhancement. This observation is further supported by the lack of a significant difference in procedure time. Our findings suggest that future investigations could explore the influence of varying compression pressures on the risk of developing larger hematomas during breast biopsy procedures. Effective breast compression pressure should be measured and analyzed in relation to hematoma size. Adjusting compression pressure may offer a promising approach to reducing hematoma formation, meriting further investigation.

The clear difference in the extent and type of angiogenesis of breast tumors^25^ is probably also reflected in our results. No significantly higher frequency of relatively larger hematomas occurred according to the different pathological findings. These potential local microvascular conditions may have little or no effect on biopsy-induced hematomas, which is supported by the lack of a significant difference in our results when epinephrine was added to the local anesthetic. Therefore, the effective benefit of the vasoconstrictive effect of local anesthetics mixed with epinephrine in MRIguided breast biopsies should be questioned^33^, as there is also a potential risk of skin necrosis.^34^

In a study by Yoen *et al*.^35^, it was observed that lesion location could influence the risk of hemorrhage, with 43% of lesions experiencing hemorrhagic complications located in the lower outer quadrant (P = 0.001). However, our findings did not reveal a significant difference in hemorrhage risk among different quadrants or in the areolar region (P = 0.399).

Additionally, our conclusions find support in the research conducted by van Deventer *et al*.^24^, who demonstrated considerable variability in the blood supply pattern of the breast through cadaveric dissections. The independent predictors identified for relatively larger hematomas, including younger age (P = 0.02), larger relative LN distances (P = 0.001), and access path length (P = 0.001), may indirectly reflect the anatomical distribution and characteristics of the perforator network relevant to our study. These perforators typically exhibit a larger diameter and closer proximity to each other in the peripheral regions of the breast near the chest wall.^24^ They radiate towards the nipple and diminish in size peripherally, consistent with their nomenclature. Consequently, hypervascular zones have been delineated by Palmer and Taylor.^24,36^ Notably, advancing age is associated with a significant risk factor for complications in perforatorpedicled propeller flap procedures in the lower extremities, particularly beyond 60 years or in the presence of known arteriopathy such as diabetes. Consequently, age-related impairments in perforator function may elucidate the significantly higher frequency of relatively larger hematomas observed in younger patients (P = 0.002). This phenomenon warrants further investigation, such as exploring MRIguided biopsies and their complications in the context of recognized arteriopathy, which could yield valuable insights. Additionally, it may be worthwhile to investigate the antero-posterior approach, which is technically feasible today, and compare it to the commonly used latero-medial approach in upright tomosynthesis-guided breast biopsies *e.g*.^37^ In conjunction with the hypervascular model of the breast proposed by Palmer and Taylor^37^, our findings underscore potential implications regarding the risk of hemorrhage.

Our study possesses several limitations that warrant acknowledgment. Firstly, it was conducted as a retrospective analysis within a single center, resulting in a relatively limited number of cases. Secondly, the classification distinguishing between relatively smaller and larger hematomas relied on the average of our hematoma results, which may not be universally applicable and could vary in larger cohorts. Thirdly, due to the inadequate number of events in the smallest outcome categories, we were unable to incorporate all investigated variables into the multivariable logistic regression analysis. Lastly, certain variables, such as operator skill, which may influence procedural outcomes, were not evaluated in this study, presenting an area for potential investigation in future research endeavours. However, we believe that the guided approach is likely to significantly reduce the impact of operator skill on procedural outcomes, making any differences minimal or negligible.

## Conclusions

Our findings underscore the importance of strategic procedural planning to minimize hematoma occurrence and enhance patient safety during MRIguided breast biopsy procedures. Understanding these factors can lead to customized approaches to improve patient outcomes and procedural efficacy.

## References

[1] Berg WA, Zhang Z, Lehrer D, Jong RA, Pisano ED, Barr RG (2012). Detection of breast cancer with addition of annual screening ultrasound or a single screening MRI to mammography in women with elevated breast cancer risk. JAMA.

[2] Kuhl C, Weigel S, Schrading S, Arand B, Bieling H, König R (2010). Prospective multicenter cohort study to refine management recommendations for women at elevated familial risk of breast cancer: the EVA trial. J Clin Oncol.

[3] La Yun B, Kim SM, Jang M, Cho N, Moon WK, Kim HH. (2016). Breast magnetic resonance imaging-guided biopsy. J Korean Soc Radiol.

[4] Zebic-Sinkovec M, Kadivec M, Podobnik G, Skof E, Snoj M. (2010). Mammographycally occult high grade ductal carcinoma (DCIS) as second primary breast cancer, detected with MRI: a case report. Radiol Oncol.

[5] Morris EA, Schwartz LH, Dershaw DD, Van Zee K, Abramson AF, Liberman L. (1997). MR imaging of the breast in patients with occult primary breast carcinoma. Radiology.

[6] Mann RM, Kuhl CK, Kinkel K, Boetes C. (2008). Breast MRI: Guidelines from the European Society of Breast Imaging. Eur Radiol.

[7] Meucci R, Pistolese Chiara A, Perretta T, Vanni G, Portarena I, Manenti G (2020). MR imaging-guided vacuum assisted breast biopsy: radiological-pathological correlation and underestimation rate in pre-surgical assessment. Eur J Radiol Open.

[8] Saslow D, Boetes C, Burke W, Harms S, Leach MO, Lehman CD (2007). American Cancer Society guidelines for breast screening with MRI as an adjunct to mammography. CA Cancer J Clin.

[9] Hoorntje LE, Peeters PH, Mali WTM, Rinkes IB. (2003). Vacuum-assisted breast biopsy: a critical review. Eur J Cancer.

[10] Borstnar S, Bozovic-Spasojevic I, Cvetanovic A, Plavetic ND, Konsoulova A, Matos E (2024). Advancing HER2-low breast cancer management: enhancing diagnosis and treatment strategies. Radiol Oncol.

[11] Zebic-Sinkovec M, Hertl K, Kadivec M, Cavlek M, Podobnik G, Snoj M. (2012). Outcome of MRIguided vacuum-assisted breast biopsy–initial experience at Institute of Oncology Ljubljana, Slovenia. Radiol Oncol.

[12] Brönnimann MP, Christe A, Heverhagen JT, Gebauer B, Auer TA, Schnapauff D (2024). Pneumothorax risk reduction during CT-guided lung biopsy–Effect of fluid application to the pleura before lung puncture and the gravitational effect of pleural pressure. Eur J Radiol.

[13] Heywang-Köbrunner SH, Sinnatamby R, Lebeau A, Lebrecht A, Britton PD, Schreer I (2009). Interdisciplinary consensus on the uses and technique of MR-guided vacuum-assisted breast biopsy (VAB): results of a European consensus meeting. Eur J Radiol.

[14] Ancona A, Caiffa L, Fazio V. (2001). [Digital stereotactic breast microbiopsy with the mammotome: study of 122 cases]. [Italian]. Radiol Med.

[15] Dershaw DD (2003). Imaging-guided interventional breast techniques.

[16] Gebauer B, Bostanjoglo M, Moesta K, Schneider W, Schlag P, Felix R. (2006). Magnetic resonance-guided biopsy of suspicious breast lesions with a handheld vacuum biopsy device. Acta Radiol.

[17] Melotti MK, Berg WA. (2000). Core needle breast biopsy in patients undergoing anticoagulation therapy: preliminary results. AJR Am J Roentgenol.

[18] Weikel W, Hofmann M, Steiner E, Bohrer M, Layer G. (2004). Stereotactic vacuum-assisted breast biopsy-analysis of 166 cases. Zentralbl Gynakol.

[19] Zagouri F, Gounaris A, Liakou P, Chrysikos D, Flessas I, Bletsa G (2011). Vacuum-assisted breast biopsy: more cores, more hematomas?. In Vivo.

[20] Brönnimann MP, Tarca M, Segger L, Kulagowska J, Florian N., Fleckenstein FN (2024). Comparative Analysis of CT Fluoroscopy Modes and Gastropexy Techniques in CT-Guided Percutaneous Radiologic Gastrostomy. Tomography.

[21] Somerville P, Seifert PJ, Destounis SV, Murphy PF, Young W. (2008). Anticoagulation and bleeding risk after core needle biopsy. AJR Am J Roentgenol.

[22] Bhat SM. (2017). SRB’s surgical operations: text & atlas.

[23] Rivard AB, Galarza-Paez L, Peterson DC. (2024). Anatomy, thorax, breast.

[24] van Deventer PV. (2004). The blood supply to the nipple-areola complex of the human mammary gland. Aesthetic Plast Surg.

[25] Boudreau N, Myers C. (2003). Breast cancer-induced angiogenesis: multiple mechanisms and the role of the microenvironment. Breast Cancer Res.

[26] Heller SL, Moy L. (2012). Imaging features and management of highrisk lesions on contrast-enhanced dynamic breast MRI. AJR Am J Roentgenol.

[27] Cohen J. (1992). Quantitative methods in psychology: A power primer. Psychol Bull.

[28] Kettritz U, Rotter K, Schreer I, Murauer M, Schulz-Wendtland R, Peter D (2004). Stereotactic vacuum-assisted breast biopsy in 2874 patients: a multicenter study. Cancer.

[29] Perlet C, Heinig A, Prat X, Casselman J, Baath L, Sittek H (2002). Multicenter study for the evaluation of a dedicated biopsy device for MR-guided vacuum biopsy of the breast. Eur Radiol.

[30] Brönnimann MP, Kulagowska J, Gebauer B, Auer TA, Collettini F, Schnapauff D (2024). Fluoroscopic-guided vs. multislice computed tomography (CT) biopsy mode-guided percutaneous radiologic gastrostomy (prg)-comparison of interventional parameters and billing. Diagnostics.

[31] Fausto A, Fanizzi A, Volterrani L, Mazzei FG, Calabrese C, Casella D (2020). Feasibility, image quality and clinical evaluation of contrast-enhanced breast MRI performed in a supine position compared to the standard prone position. Cancers.

[32] McGrath AL, Price ER, Eby PR, Rahbar H. (2017). MRIguided breast interventions. J Magn Reson Imaging.

[33] Flowers CI. (2012). Breast biopsy: anesthesia, bleeding prevention, representative sampling, and rad/path concordance. Applied Radiology.

[34] Hartzell TL, Sangji NF, Hertl MC. (2010). Ischemia of postmastectomy skin after infiltration of local anesthetic with epinephrine: a case report and review of the literature. Aesthetic Plast Surg.

[35] Yoen H, Chung HA, Lee SM, Kim ES, Moon WK, Ha SM. (2024). Hemorrhagic complications following ultrasound-guided breast biopsy: A prospective patientcentered study. Korean J Radiol.

[36] Palmer JH, Taylor GI. (1986). The vascular territories of the anterior chest wall. Br J Plast Surg.

[37] Vijapura CA, Wahab RA, Thakore AG, Mahoney MC. (2021). Upright tomosynthesis-guided breast biopsy: Tips, tricks, and troubleshooting. Radiographics.

